# ISCB Computational Biology Wikipedia Competition

**DOI:** 10.1371/journal.pcbi.1003242

**Published:** 2013-09-19

**Authors:** Alex Bateman, Janet Kelso, Daniel Mietchen, Geoff Macintyre, Tomás Di Domenico, Thomas Abeel, Darren W. Logan, Predrag Radivojac, Burkhard Rost

**Affiliations:** 1 European Molecular Biology Laboratory, European Bioinformatics Institute, Hinxton, United Kingdom; 2 Max Planck Institute for Evolutionary Anthropology, Leipzig, Germany; 3 Museum für Naturkunde - Leibniz-Institut für Evolutions- und Biodiversitätsforschung, Berlin, Germany; 4 NICTA Victoria Research Laboratory, Department of Electrical and Electronic Engineering, University of Melbourne, Victoria, Australia; 5 Department of Biology, University of Padua, Padova, Italy; 6 Broad Institute of MIT and Harvard, Genome Sequencing and Analysis Program, Cambridge, Massachusetts, United States of America; 7 VIB Department of Plant Systems Biology, Ghent University, Ghent, Belgium; 8 Wellcome Trust Sanger Institute, Wellcome Trust Genome Campus, Hinxton, United Kingdom; 9 School of Informatics and Computing, Indiana University, Bloomington, Indiana, United States of America; 10 Technische Universität München, München, Germany

The International Society for Computational Biology is pleased to announce the 2013 ISCB Computational Biology Wikipedia competition. The competition, in which entrants create or improve the content of any Wikipedia article in the field of computational biology, is open to all students and trainees. Further information about the competition can be found here: http://en.wikipedia.org/wiki/Wikipedia:WikiProject_Computational_Biology/ISCB_competition_announcement_2013

The mission of the ISCB is to promote the use of computational biology and to help educate the next generation of computational biologists. The society has numerous activities that help to address these aims, including conferences, training and mentoring initiatives, and an active student council.

As the world's largest online encyclopedia, Wikipedia has become an indispensable resource for those seeking information on all scientific and technical topics. The English language version of Wikipedia contains over 4.2 million articles, and Wikipedia is now available in 286 languages. The global rise in smartphone use, which allows access to Wikipedia, means that a large fraction of the world's population can now gain access to the world's knowledge. Wikipedia is the most successful example of crowd-sourcing with about 80,000 active editors updating its content each month.

But is Wikipedia a good source of information for computational biology? Certainly, many people are reading the articles. For example, the Bioinformatics article has been visited 1,600 times per day over the last 3 months. Wikipedia contains articles on algorithms, biological databases, software packages, and biographies of eminent computational biologists. The computational biology content ranges from incomplete, a mere “stub” of an article in Wikipedia parlance, to highly detailed Featured Articles. A group of Wikipedia editors have formed the Computational Biology Wikiproject (http://en.wikipedia.org/wiki/Wikipedia:WikiProject_Computational_Biology). This group oversees the computational biology articles and rates them for their importance and their quality. Figure 1 shows the current state of the articles (see also Figure S1). In total, there are over 1,140 articles that have been considered as falling under Computational Biology. There are a small number of articles that have been brought up to the highest levels of quality (Featured Article and Good Article) such as Multiple Sequence Alignment, Genome Wide Association Study, and Folding@home.

**Figure 1:**
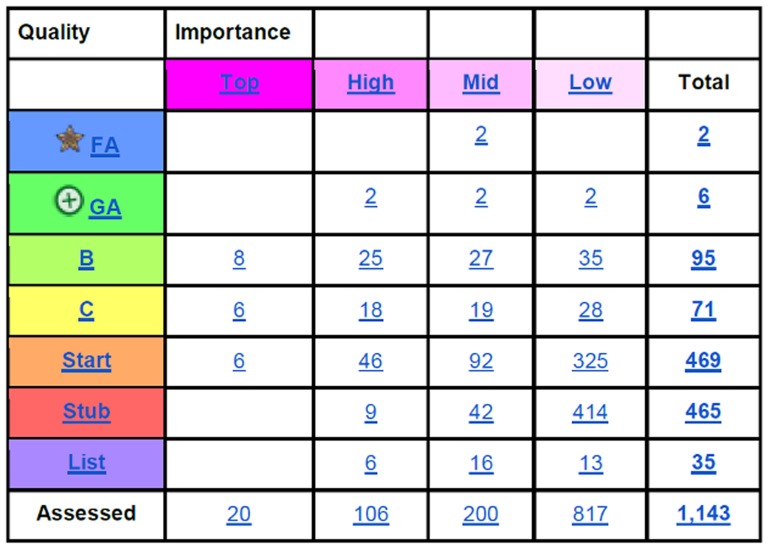
The computational biology articles rated by quality and importance by the Wikipedia Computational Biology Wikiproject. The quality levels go from the best to the lowest, in the following order: FA (Featured Article), GA (Good Article), B-class, C-class, Start, Stub. Some articles are simply lists, and these are not rated for quality. See Figure S1 for a version that includes hyperlinks.

The 2012 competition began 9th September 2012 (coinciding with the start of the European Conference on Computational Biology) and finished four months later on the 10th January 2013. Each article entered in the competition was reviewed for a difference in article quality between these two dates. In 2012, there were 13 substantive entries into the competition. Six of these articles were shortlisted by members of the ISCB Student Council and then considered by the judging panel. The judging panel considered articles based on the criteria of clarity of the writing, depth of knowledge of the subject, and quality of figures and images used. In one case, it was clear that the article was largely derived from a published review, and was not considered further. For the other entries, the quantity and quality of the contributions were very good, and it was a challenge to rank the articles. After much deliberation, the judging panel selected the following as the winners of the 2012 ISCB Wikipedia competition:

1st prize: James Estevez for improvements to the Genomics Article.
2nd prize: Benjamin Moore for improvements to the European Nucleotide Archive article.
3rd prize: Luis Pedro Coelho for improvements to the Bioimage Analysis article.

We are keen to grow the depth and quality of computational biology articles and wish to encourage the widest possible range of students and trainees to take part. We envisage that teachers, tutors, and lecturers could use the competition as an opportunity to train students in literature research on topics of computational biology. This approach to literature review provides the students with a thorough grounding in the subject area of the article. In addition, the collaborative writing environment of Wikipedia encourages critical thinking and improves literature research skills. Furthermore, compared to traditional literature reviews carried out by students, which typically end up unread in a filing cabinet, contributing to Wikipedia means that the students' scholarly contributions will be publicly visible.

We hope that the ISCB Wikipedia competition will continue to grow and help improve the quality of Computational Biology information freely available on the Internet. We are interested in improving not just the articles in Wikipedia, but also the associated media, such as images and figures on Wikimedia Commons, and data through Wikidata. We encourage you to get involved by either entering the competition if you are a student or trainee, or getting your own students to participate.

## About ISCB

The International Society for Computational Biology (ISCB) - www.iscb.org - is the first, and continues to be the only, society representing computational biology and bioinformatics on a worldwide scale. ISCB serves a global community of over 3,000 scientists dedicated to advancing the scientific understanding of living systems through computation. It convenes the world's experts and future leaders in top conferences and partners with publications that promote discovery and expand access to computational biology and bioinformatics. It delivers valuable information about training, education, employment, and relevant news. ISCB also provides an influential voice on government and scientific policies that are important to its members and benefit the public.

The ISCB is incorporated in the United States as a 501(c) (3) non-profit corporation, and registered in the state of California as a Charitable Trust. For more information about ISCB and its initiatives and programs, please visit www.iscb.org.

## Supporting Information

Figure S1 **The computational biology articles rated by quality and importance by the Wikipedia Computational Biology Wikiproject.** The quality levels go from the best to the lowest, in the following order: FA (Featured Article), GA (Good Article), B-class, C-class, Start, Stub. Some articles are simply lists, and these are not rated for quality. This is a version of Figure 1 with hyperlinks.
(PDF)

