# Supplementary material for: ISCB Computational Biology Wikipedia Competition
Source: PLoS Comput Biol. 2013 Sep 19;9(9):e1003242. doi: 10.1371/journal.pcbi.1003242 (PMC3777890; doi:10.1371/journal.pcbi.1003242)
Supplement: Figure S1 — The computational biology articles rated by quality and importance by the Wikipedia Computational Biology Wikiproject. The quality levels go from the best to the lowest, in the following order: FA (Featured Article), GA (Good Article), B-class, C-class, Start, Stub. Some articles are simply lists, and these are not rated for quality. This is a version of Figure 1 with hyperlinks. (PDF) [file pcbi.1003242.s001.pdf]

Quality	Importance				
	Top	High	Mid	Low	Total
FA			2		**2**
GA		2	2	2	**6**
B	8	25	27	35	**95**
C	6	18	19	28	**71**
Start	6	46	92	325	**469**
Stub		9	42	414	**465**
List		6	16	13	**35**
**Assessed**	20	106	200	817	**1,143**
